# L'atteinte oculaire au cours de l'hypertension artérielle maligne

**DOI:** 10.11604/pamj.2014.17.9.3804

**Published:** 2014-01-14

**Authors:** Zouheir Hafidi, Rajae Daoudi

**Affiliations:** 1 Université Mohammed V Souissi, Service d'Ophtalmologie A de l'hôpital des spécialités, Centre Hospitalier Universitaire, Rabat, Maroc

**Keywords:** Atteinte oculaire, hypertension artérielle maligne, œdème papillaire, fond d'oeil, Ocular involvement, malignant hypertension, papilledema, fundus

## Image en médicine

Une patiente âgée de 55 ans suivie pour une hypertension artérielle (mauvaise observance) est admise pour une baisse brutale d'acuité visuelle dans un contexte de céphalées et des acouphènes. A l'examen l'acuité visuelle était de 2/10e au niveau de l'oeil droit et compte les doigts à 1 mètre au niveau de l'oeil gauche, l'examen du fond d'oeil révèle un oedème papillaire bilatéral plus marqué à gauche avec des hémorragies en flammèches et des exsudats secs, par ailleurs il y avait un rétrécissement marqué de tout l'arbre vasculaires artériel. Le reste de l'examen général a mis en évidence une TA de 220mmhg de systolique et 110mmgh de diastolique. Après rééquilibration rapide de la TA un bilan a été réalisé à savoir: urée, créatinine dans le sang, ionogramme sanguin complet qui n'a pas révélé d'anomalies. Une TDM cérébrale a permis d’éliminer un processus expansif intracrânien, une hydrocéphalie et une thrombose veineuse. La patiente fut alors adressée au département de cardiologie pour complément de prise en charge. L'angiographie rétinienne a mis en évidence une forte rétention papillaire de la fluorescéine avec des altérations diffusantes de tout le lit capillaire rétinien en rapport avec l'altération de la barrière hémato rétinienne. L'Hypertension artérielle maligne correspond à une augmentation rapide et sévère des chiffres tensionnels. Cette entité concerne moins de 1% des patients hypertendus et constitue une urgence vitale par atteinte des organes cibles (cerveau, oeil, coeur, reins) avec un risque important de: AVC hémorragique, IDM, insuffisance rénale. Sur le plan ophtalmologique, et en l'absence d'une prise en charge rapide, il existe un risque important d'atrophie optique avec perte visuelle définitive secondaire à une neuropathie optique ischémique.

**Figure 1:**
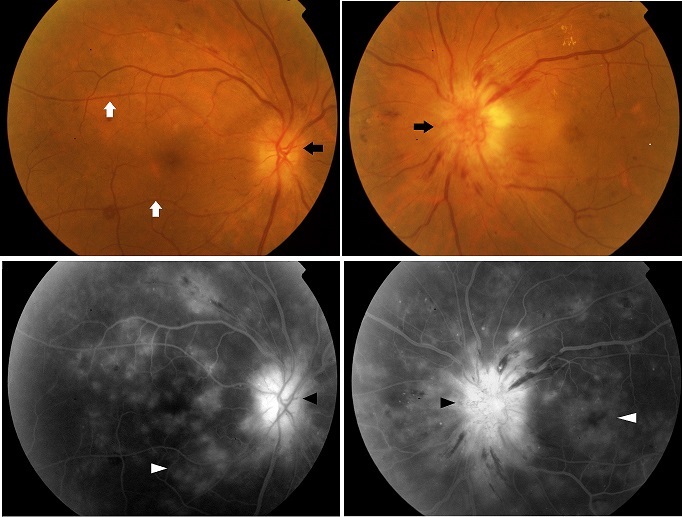
(En haut) examen du Fond d’œil mettant en évidence un œdème papillaire bilatéral (flèches noires) plus marqué à gauche associé à de multiples taches hémorragiques avec un rétrécissement artériel manifeste (flèches blanches); (en bas) angiographie à la fluorescéine confirmant l’œdème papillaire (rétention de la fluorescéine, têtes de flèches noires) et mettant en évidence des altérations diffusantes de tout le lit capillaire rétinien (diffusion de la fluorescéine, têtes de flèches blanches).

